# AMBRA1 Inhibits Non‐Small Cell Lung Cancer Progression Through miR‐1178/p53/CDK2‐Regulated Cell Cycle Arrest

**DOI:** 10.1111/jcmm.70610

**Published:** 2025-06-04

**Authors:** Jing Feng, Shan Li, Laihua Li, Zhiqiang Du, Guangying Yang, Zhi Zhao, Xueke Fan, Na Wang, Zhigang Zhao

**Affiliations:** ^1^ Zhengzhou Yihe Hospital Affiliated to Henan University Zhengzhou Henan Province China; ^2^ Henan University of Chinese Medicine Zhengzhou Henan Province China

**Keywords:** AMBRA1, CDK2, cell cycle, microRNA‐1178, NSCLC, p53

## Abstract

AMBRA1 is associated with a variety of pathological processes in cancer cells, but may have different functions in different tumour microenvironments or genetic backgrounds. In this study, the function and regulatory mechanisms of AMBRA1 were explored in the progression of non‐small cell lung cancer (NSCLC). The abnormally expressed miRNAs in AMBRA1‐overexpressed and differentially expressed genes in miR‐1178‐knockdown NSCLC cells were validated by RNA sequencing. Cell viability, proliferation, invasion, apoptosis, and cell cycle were tested through Cell Counting Kit‐8 (CCK‐8), EdU, colony formation, transwell, and flow cytometry. A mouse tumour xenograft model was conducted to assess the roles of the AMBRA1‐miR‐1178 axis on NSCLC progression in vivo. AMBRA1 overexpression suppressed NSCLC cell proliferation and invasion, while promoting apoptosis and G0/G1 phase cell cycle arrest in vitro, and inhibited tumour growth in vivo. RNA sequencing revealed miR‐1178 as a target of AMBRA1. miR‐1178 overexpression partially weakened the suppressive function of AMBRA1 on cell malignant biological behaviours. p53 and CDK2 were confirmed as the downstream targets of miR‐1178. Silencing p53 or overexpressing CDK2 reversed the repressive effects of AMBRA1 on the development of NSCLC cells. AMBRA1 may suppress the malignant phenotype of NSCLC cells via regulating the miR‐1178‐p53‐CDK2 signalling pathway.

**Abbreviations**
ActD: actinomycin D
AMBRA1: activating molecule in Beclin‐1‐regulated autophagy protein 1
EMT: epithelial‐mesenchymal transition
HCC: hepatocellular carcinoma
LUAD: lung adenocarcinoma
miRNAs: microRNAs
NSCLC: non‐small cell lung cancer

## 1 Introduction

Lung cancer has the highest incidence and mortality rate in the world, among which non‐small cell lung cancer (NSCLC) constitutes about 80% of lung cancer cases [1, 2]. Although great strides have been made in conventional treatments such as surgical resection and adjuvant chemotherapy, due to recurrence and metastasis, NSCLC patients still have a poor prognosis [3, 4]. Therefore, NSCLC patients' 5‐year survival rate is only 13% [5]. With recent advances in sequencing technology, individualised treatment characterised by genetic diagnosis and molecular targeted therapy has become a promising treatment for NSCLC [6, 7]. This highlights the urgent need to find new molecular targets and determine the relevant mechanism of action for the early diagnosis and treatment of NSCLC.

Activating molecule in Beclin‐1‐regulated autophagy protein 1 (AMBRA1) is a pro‐autophagy protein and is also involved in the regulation of apoptosis. AMBRA1 decides cell survival or death by maintaining a balance between apoptosis and autophagy and has been shown to be implicated in modulating the sensitivity of cancer cells to anticancer drugs [8, 9, 10]. Besides, AMBRA1 is the main regulator of D‐type cyclins degradation and an upstream main regulator transitioning from G1 to S phase; therefore, it is a participant in regulating cell cycle in tumour cells [11, 12, 13]. Furthermore, several researches have also shown that AMBRA1 participates in the modulation of tumour cell migration and invasion via epithelial‐mesenchymal transition (EMT) [14]. Therefore, AMBRA1 participates in tumorigenesis and progression by regulating tumour cell autophagy, apoptosis, cell cycle, proliferation, and metastasis.

As small single‐stranded non‐coding RNAs of about 22 nucleotides in length, MicroRNAs (miRNAs) typically inhibit protein synthesis via base‐pairing with the 3′‐UTR of mRNAs, thereby post‐transcriptionally modulating gene expression [15]. It has been verified that miRNAs play multiple roles in the development and progression of NSCLC and are attractive targets for novel therapeutic approaches for NSCLC [16]. However, Li et al. analysed four independent databases and identified that only six miRNAs (miR‐200b‐3p, miR‐200c‐3p, miR‐23a‐3p, miR‐429, miR‐7‐5p and miR‐9‐5p) could target AMBRA1, indicating that the number of conserved miRNA binding sites in AMBRA1 3′‐UTR is limited, suggesting these miRNAs might be involved in regulating cancer proliferation and chemosensitivity by targeting AMBRA1 [17]. More importantly, previous studies have proved that MIR7‐3HG [18], miR‐200b [19], miR‐3653 [20] and miRNA‐198 [21] directly targeted AMBRA1. In which, MIR7‐3HG was found to target and downregulate AMBRA1 expression to prevent the dephosphorylation and degradation of MYC, thereby enhancing its own transcription level and promoting NSCLC A549 cell proliferation [18]. And miR‐3653 inhibited the autophagy process of breast cancer cells by targeting AMBRA1, thereby inhibiting EMT and metastasis [20]. As miRNAs are highly tissue‐specific, they can be employed for the prediction of cancer molecular phenotype; these specific miRNAs may serve as a fundamental method for the diagnosis and management of cancers with AMBRA1 abnormalities.

In this research, we verified the function of AMBRA1 in the growth and metastasis of NSCLC, and performed RNA sequencing to find differentially expressed miRNAs after abnormal expression of AMBRA1. Our study results might offer a new theoretical foundation for the development of targeted clinical treatments for NSCLC.

## 2 Materials and Methods

### 2.1 Cell Culture and Transfection

NSCLC cell lines (A549 and H1299) were purchased from the ATCC (VA, USA) and were cultured at 37°C with 5% CO_2_ in DMEM (Gibco, NY, USA) supplemented with 10% FBS (Gibco), 100 U/mL penicillin, and 0.1 mg/mL streptomycin (Gibco).

The design and synthesis of two shRNA targeting AMBRA1 (AMBRA1‐shKD‐1 and AMBRA1‐shKD‐2) and scrambled negative control (AMBRA1‐shNC) were provided by RiboBio (Guangzhou, China). To overexpress AMBRA1 (AMBRA1‐oe) and CDK2 (CDK2‐oe), the cDNA of AMBRA1 and CDK2 was synthesised and cloned into the pcDNA3.1 vector, and the empty plasmid was utilised as the negative control (AMBRA1‐vector and CDK2‐vector). siRNA targeting p53 (p53‐siKD) and negative control (p53‐siNC), miR‐1178 mimic/inhibitor (miR‐1178‐oe/‐siKD) and mimic/inhibitor NC (miR‐1178‐vector/‐siNC) were provided by Sangon Biotech (Shanghai, China). Next, the above shRNAs/siRNAs/vectors/mimics/inhibitor were delivered to NSCLC cells utilising Lipofectamine 3000 reagent (Invitrogen, CA, USA). Cells were harvested 48 h later for subsequent experiments.

### 2.2 qRT‐PCR

Total RNAs were isolated from NSCLC cells utilising the TRIzol reagent (Invitrogen). The generation of cDNA was performed utilising the PrimeScript RT Reagent Kit (TaKaRa, Japan). Next, SYBR Premix Ex Taq II (TaKaRa) was applied to quantify mRNA and miRNA in a Thermal Cycler CFX6 System (Bio‐Rad, CA, USA). With GAPDH as the internal reference for AMBRA1, p53, and CDK2 and U6 for miR‐1178, the relative mRNA and miRNA levels were calculated by the 2^−ΔΔ*Ct*^ method.

### 2.3 Western Blot

Isolating total proteins from NSCLC cells and mice tumour tissues utilised RIPA lysis buffer (Beyotime, Shanghai, China). The proteins were transferred to PVDF membranes (Millipore, MA, USA) after separation by SDS‐PAGE. Next, the membrane was blocked with skim milk, hybridised with the primary antibody against AMBRA1, p53, CDK2, PLIN5, APOA4, TMEM174, DUSP8, CAS3, GSTT4, or GAPDH (Abcam, MA, USA) overnight at 4°C. Thereafter, incubation of the membrane with the secondary antibody was carried out for 2 h at room temperature. The bands were visualised by interacting with the ECL reagent (Invitrogen) and analysed with ImageJ software.

### 2.4 CCK‐8 Assay

NSCLC cells (2 × 10^3^ cells/well) were seeded into 96‐well plates. CCK‐8 solution (10 μL/well, Dojindo, Japan) was added to the culture medium after 24 h incubation and incubated for an additional 4 h. Finally, the optical density (OD) at 450 nm was observed at different times (0, 24, 48, 72 and 96 h) using a microplate reader (Bio‐Rad).

### 2.5 EdU Assay

Transfected NSCLC cells (1 × 10^4^ cells/well) were cultured in 96‐well plates and incubated with 100 μL 50 μM EdU solution (Ribobio)‐containing medium for 2 h at 37°C. The cultured cells were subsequently fixed for 30 min in 4% formaldehyde and permeabilized for 20 min with 0.1% Triton X‐100. After washing with PBS, they were stained with EdU staining cocktail for 30 min at room temperature, avoiding light, and the cell nucleus was counterstained with DAPI. Stained cells were visualised by fluorescence microscopy (Nikon, Japan).

### 2.6 Transwell Assay

Matrigel‐coated Transwell chambers (Corning, NY, USA) were utilised to measure cell invasion. 500 μL of complete DMEM medium was put into the lower chamber. The upper chamber was introduced with transfected NSCLC cells (2 × 10^4^ cells). Following a 24 h incubation, cells attached to the lower surface were fixed with methanol for 10 min and stained for 15 min with 0.1% crystal violet. Finally, under the microscope (Olympus, Japan), the NSCLC cell invasion was examined.

### 2.7 Colony Formation Assay

The transfected NSCLC cells (500 cells/well) were evenly dispersed in 6‐well plates and incubated for 2 weeks. Afterwards, with 4% paraformaldehyde, the cells were fixed and followed by staining with 0.1% crystal violet for 30 min. Colonies were visible and counted by microscopy (Olympus) and the relative number of colonies was calculated. All colony formation assays were performed under identical conditions.

### 2.8 Flow Cytometry

For cell apoptosis, the transfected NSCLC cells were re‐suspended in 400 μL of 1× binding buffer and stained with 10 μL PI and 5 μL Annexin V‐FITC using an FITC Annexin V Apoptosis Detection kit (Beyotime) at 4°C for 15 min avoiding light. Eventually, the percentage of apoptotic cells was analysed utilising FACSCalibur (BD Biosciences, NJ, USA).

For cell cycle assessment, the transfected NSCLC cells were harvested, then fixed in ice‐cold ethanol and incubated for 30 min. Next, cells were subjected to RNase (Sigma‐Aldrich, MO, USA) treatment followed by 30 min of cell staining with PI. Alternatively, DNA was stained using a solution containing RNase and Click‐iT EdU CellCycle Dye (Invitrogen, OR, USA). Finally, analysing cell cycle distribution with FACSCalibur (BD Biosciences).

### 2.9 RNA Sequencing and Analysis

The total RNA was extracted from the AMBRA1‐vector, AMBRA1‐oe, miR‐1178‐siNC, and miR‐1178‐siKD H1299 cells by using the TRIzol reagent (Invitrogen). RNA sequencing was performed by Novogene (Beijing, China). log2 (fold change) > 1 and an adjusted *p* value < 0.05 were regarded as thresholds for differentially expressed genes (DEGs). The heat map and volcano plot of DEGs were plotted with R software.

### 2.10 Half‐Life Analysis

H1299 cells (5 × 10^5^ cells/well) were cultured in 6‐well plates for 24 h. Next, 5 μg/mL actinomycin D (ActD, Sigma‐Aldrich) was utilised for cell treatment. At indicated time points, cells were collected and the stability of RNA was determined by qRT‐PCR.

### 2.11 Tumour Xenograft Model

For xenograft experiments, 5‐week‐old BALB/c nude mice (Vital River Laboratory, Beijing, China) were used. H1299 cells (2 × 10^6^ cells) transfected with AMBR1‐vector or AMBR1‐oe and miR‐1178‐oe were injected subcutaneously into the right side of each mouse. Tumour length and width were recorded every 3 days and calculated according to the formula volume = length × width^2^ × 0.5. On day 21, mice were sacrificed, tumours were excised, imaged, and weighed. The animal experiment was approved by the Animal Ethics Committee of Zhengzhou Yihe Hospital Affiliated to Henan University.

### 2.12 TUNEL Assay

A 10% formaldehyde solution was applied to fix the tumour samples and embedded in paraffin. Tumour tissues were subsequently sectioned and dehydrated in graded concentrations of ethanol and cleared in xylene. Tissue sections were then incubated with proteinase K for 20 min at 37°C. After washing 3 times with PBS buffer, the slices were incubated in 1× equilibration buffer for 30 min, then 50 μL TUNEL reaction mixture was added and hatched for 1 h at 37°C away from light. Subsequently, the sections were washed 3 times with PBS buffer, and then DAPI staining solution was added and incubated for 5 min in darkness. Slides were observed by a fluorescence microscope (Nikon).

### 2.13 Statistical Analysis

Results were expressed as mean ± standard deviation (SD). Statistical analyses were conducted utilising GraphPad Prism 8 software, including Student's t‐test when comparing two experimental groups and ANOVA for more than two groups. *p* < 0.05 was considered statistically significant.

## 3 Results

### 3.1 AMBRA1 Suppressed Proliferation and Metastasis of NSCLC Cells

**FIGURE 1:**
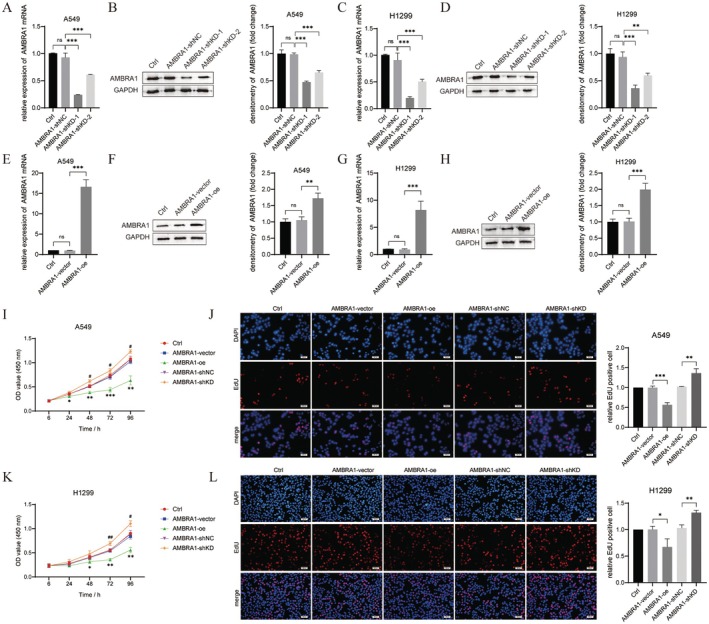
AMBRA1 overexpression inhibited NSCLC cell proliferation. (A–D) Interference efficiency of two alternative shRNAs targeting AMBRA1 was confirmed by qRT‐PCR and western blot in A549 and H1299 cells. The bar graph on the right shows the quantification of the grey values of the protein bands. (E–H) Overexpression efficiency of AMBRA1‐oe was tested by qRT‐PCR and western blot in A549 and H1299 cells. (I–L) Cell viability and proliferation were tested by CCK‐8 and EdU assays in A549 and H1299 cells transfected with AMBRA1‐shKD or AMBRA1‐oe. The amount of CCK‐8 experimental cells was 2 × 10^3^ cells/well and the amount of EDU experimental cells was 1 × 10^4^ cells/well. Data are representative of three independent experiments (*n* = 3, mean ± SD). *p < 0.05, **p < 0.01 and ***p < 0.001 versus indicated group or AMBRA1‐vector; #*p* < 0.05 and ##*p* < 0.01 versus AMBRA1‐shNC.

**FIGURE 2:**
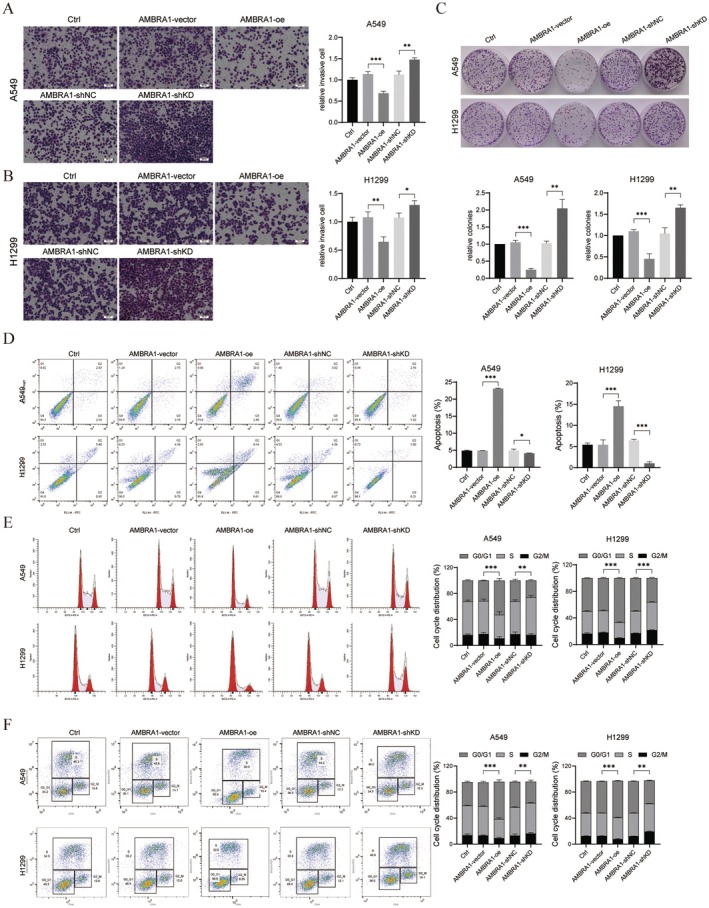
AMBRA1 overexpression suppressed the invasion while increased apoptosis and G0/G1 phase‐cell cycle arrest of NSCLC cells. A549 (A) and H1299 (B) cells transfected with AMBRA1‐shKD or AMBRA1‐oe were seeded into Matrigel‐coated chambers, and cell invasion was tested by transwell assay, and counted in 3 random fields per sample. (C) The number of colonies formed was determined by colony formation assay; for A549 cells, the number of colonies in the AMBRA1‐vector group was 422, in the AMBRA1‐oe was 219, in the AMBRA1‐shNC was 428, and in the AMBRA1‐shKD was 489. As for H1299 cells, the vector group formed 447 colonies, the AMBRA1‐oe formed 198 colonies, the AMBRA1‐shNC formed 421 colonies, and the AMBRA1‐shKD formed 500 colonies. Cells were stained with Annexin V‐FITC/PI and the percentage of apoptotic cells was analysed by flow cytometry (D). Stained with PI (E) and Click‐iT EdU (F) and analysed by flow cytometry. G0/G1, S, and G2/M phase percentages. Results are shown as mean ± SD of three biological replicates. *p < 0.05, **p < 0.01 and ***p < 0.001 versus indicated group.

First, A549 and H1299 cells were transfected with AMBRA1‐shKD (−1 and − 2) and AMBRA1‐oe respectively to down‐regulate and overexpress AMBRA1. Transfection efficiency was validated by qRT‐PCR and western blot (*p* < 0.01 and *p* < 0.001, Figure 1A–H). Further CCK‐8 and EdU assays indicated that A549 and H1299 cell viability and proliferation were suppressed by AMBRA1 overexpression (*p* < 0.05, *p* < 0.01 and *p* < 0.001), while they were promoted by silencing AMBRA1 (*p* < 0.05 and *p* < 0.01, Figure 1I–L). Additionally, transwell results showed that A549 and H1299 cell invasive ability was weakened by AMBRA1 overexpression (*p* < 0.01 and *p* < 0.001), whereas it was enhanced by AMBRA1 silencing (*p* < 0.05 and *p* < 0.01, Figure 2A,B). Likewise, colony formation assay demonstrated AMBRA1 overexpression decreased A549 and H1299 colony formation (*p* < 0.001), whereas AMBRA1 silencing increased colony formation (*p* < 0.01, Figure 2C). For cell apoptosis assessment, as determined by flow cytometry, AMBRA1 overexpression increased the rate of apoptosis in A549 and H1299 cells (*p* < 0.001), whereas AMBRA1 silencing suppressed cell apoptosis (*p* < 0.05 and *p* < 0.001, Figure 2D). Cell cycle distribution of A549 and H1299 cells was tested by flow cytometry as well. As shown in Figure 2E, AMBRA1 overexpression increased G0/G1‐phase cell population (*p* < 0.001), while AMBRA1 silencing had the opposite result (*p* < 0.01 and *p* < 0.001), while Figure 2F shows superimposed DNA histograms of the G1, S and G2/M phases of the cell cycle, which indicated AMBRA1 expression contributed to G0/G1 phase arrest of NSCLC cells.

### 3.2 AMBRA1 Overexpression Inhibited NSCLC Cell Proliferation and Invasion Through Decreasing miR‐1178 Expression

**FIGURE 3:**
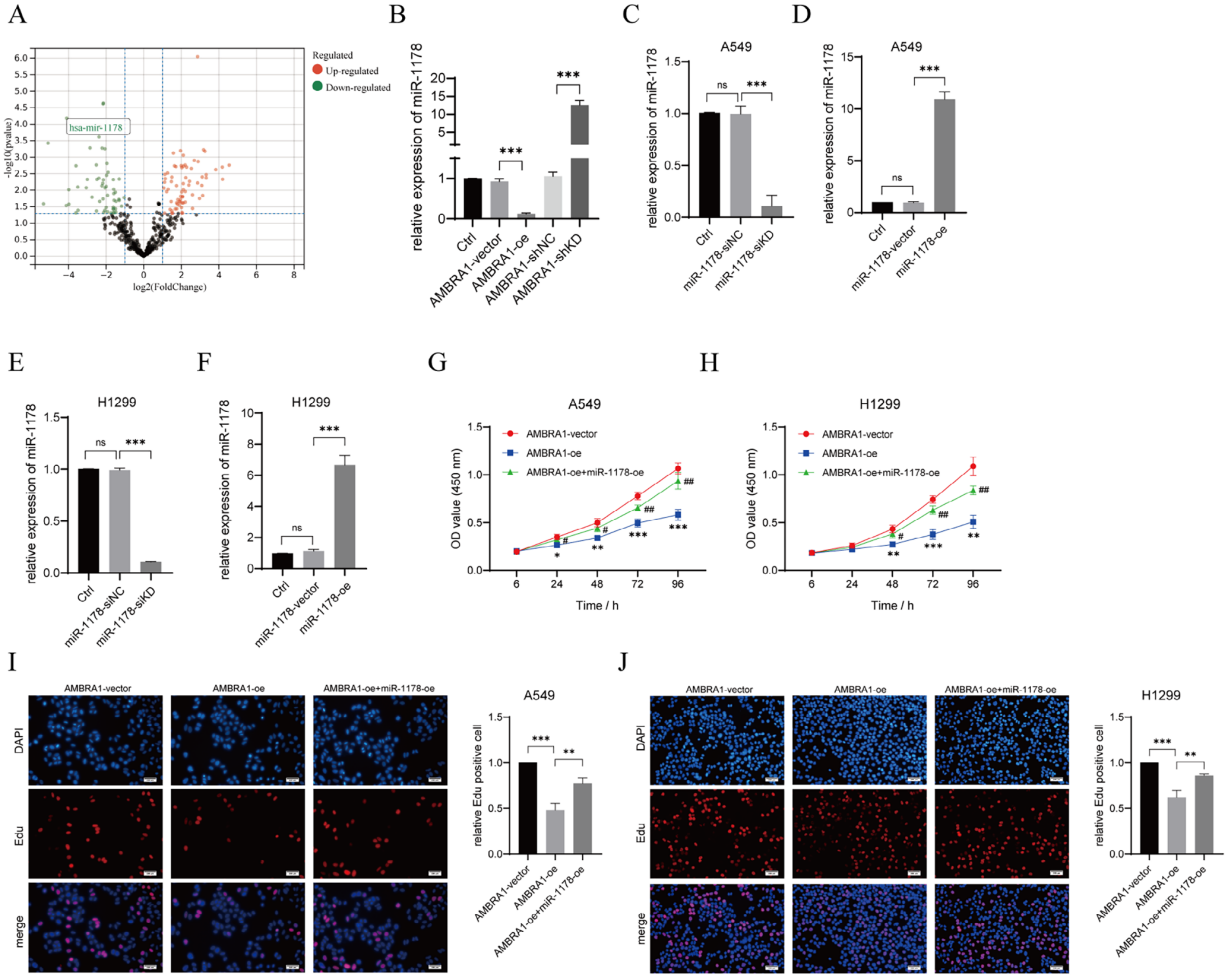
AMBRA1 overexpression suppressed NSCLC cell proliferation through down‐regulating miR‐1178. (A) Volcano plots of differentially expressed miRNAs in AMBRA1‐overexpressed H1299 cells. Green points, downregulated miRNAs; red points, upregulated miRNAs in AMBRA1‐overexpressed H1299 cells; black points, miRNAs without significant changes. (B) miR‐1178 level in AMBRA1‐overexpressed or AMBRA1‐knockdown H1299 cells by qRT‐PCR. (C–F) Transfection efficiency of miR‐1178‐siKD and miR‐1178‐oe was verified by qRT‐PCR in A549 and H1299 cells. (G–J) Cell viability and proliferation were tested by CCK‐8 and EdU assays in A549 and H1299 cells co‐transfected with AMBRA1‐oe and miR‐1178‐oe. Results are shown as mean ± SD of three biological replicates. *p < 0.05, **p < 0.01 and ***p < 0.001 versus indicated group or AMBRA1‐vector; #*p* < 0.05 and ##*p* < 0.01 versus AMBRA1‐oe group.

Differentially expressed miRNAs were analysed in AMBRA1‐overexpressed H1299 cells, as presented in Figure 3A. miR‐1178 was the most significantly downregulated miRNA in AMBRA1‐overexpressed H1299 cells. Further, the miR‐1178 expression was verified by qRT‐PCR; miR‐1178 was found to be upregulated in AMBRA1‐knockdown H1299 (*p* < 0.001), whereas it was down‐regulated after AMBRA1 overexpression (*p* < 0.001, Figure 3B). The above results suggested that AMBRA1 negatively modulated the miR‐1178 expression in NSCLC cells. Therefore, we next verified the role of miR‐1178 in AMBRA1‐suppressed NSCLC cell malignant phenotypes.

**FIGURE 4:**
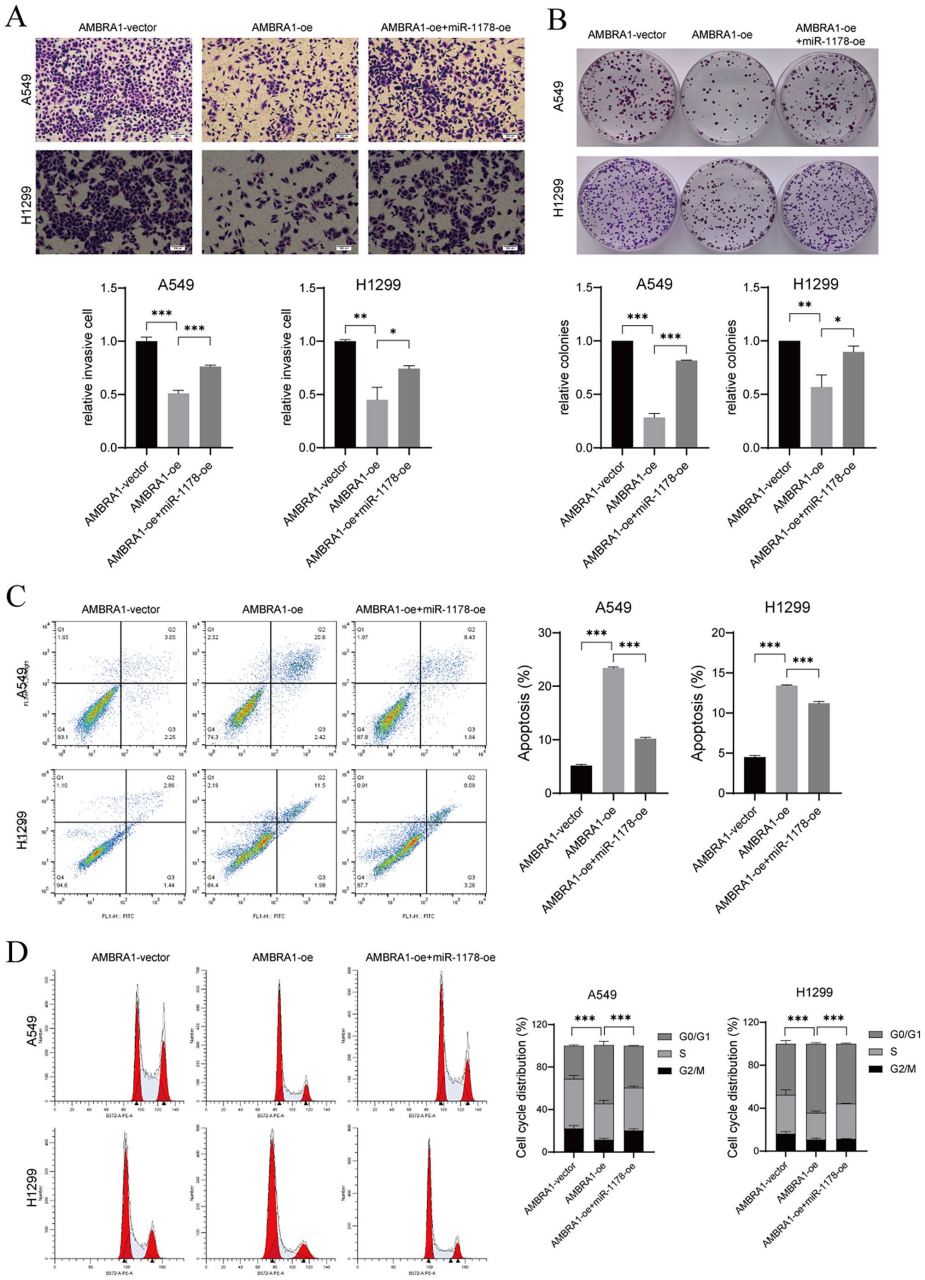
AMBRA1 overexpression inhibited NSCLC cell invasion through decreasing miR‐1178 expression. A549 and H1299 cells were co‐transfected with AMBRA1‐oe and miR‐1178‐oe. (A) cell invasive was tested by transwell assay. (B) colony formation assay was conducted to determine cell proliferation. For A549 cells, the number of colonies in the AMBRA1‐vector group was 376, in the AMBRA1‐oe was 177, and in the AMBRA1‐oe + miR‐1178‐oe was 334. As for H1299 cells, the vector group formed 480 colonies, the AMBRA1‐oe formed 312 colonies, and the AMBRA1‐oe + miR‐1178‐oe formed 435 colonies. The apoptotic cell rate (C) and cell cycle distribution (D) were verified by flow cytometry. Results are shown as mean ± SD of three biological replicates. *p < 0.05, **p < 0.01 and ***p < 0.001 versus indicated group.

First, we suppressed or overexpressed miR‐1178 in A549 and H1299 cells and validated that we successfully constructed miR‐1178‐knockdown or ‐overexpressed A549 and H1299 cells by qRT‐PCR (*p* < 0.001, Figure 3C–F). MiR‐1178 overexpression partly counteracted the suppressive effects of AMBRA1overexpression on A549 and H1299 cell viability, proliferation, and invasion (*p* < 0.05, *p* < 0.01 and *p* < 0.001, Figures 3G and 4B). AMBRA1 overexpression also promoted A549 and H1299 cell apoptosis and G0/G1 phase cell arrest, partly achieved by decreasing the expression of miR‐1178 (*p* < 0.001, Figure 4C,D).

### 3.3 miR‐1178 Regulated p53/CDK2 Axis in NSCLC Cells

**FIGURE 5:**
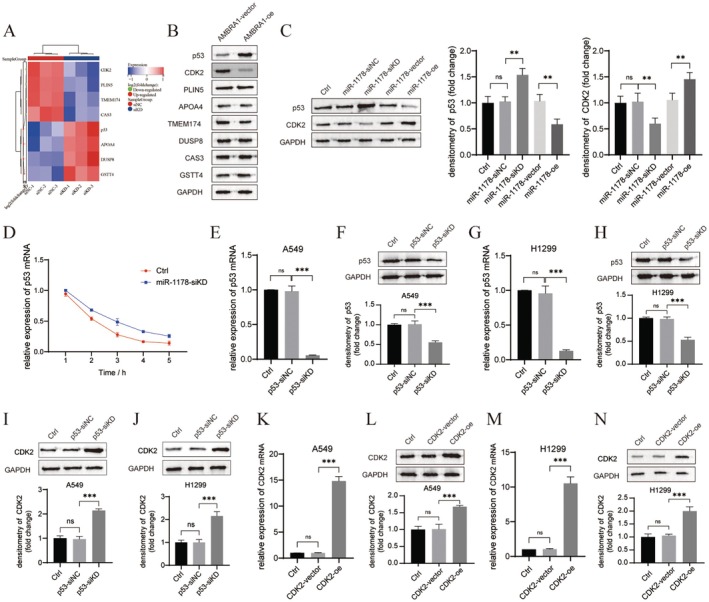
miR‐1178 regulated p53/CDK2 expression in NSCLC cells. (A) Heatmap of DEGs in miR‐1178‐silenced H1299 cells. Green points, downregulated DEGs; red points, upregulated DEGs in miR‐1178‐silenced H1299 cells. (B) Western blot results of DEGs in AMBRA1‐overexpressed H1299 cells. (C) p53 and CDK2 protein expression in miR‐1178‐silenced or miR‐1178‐overexpressed H1299 cells was confirmed by western blot. (D) H1299 cells transfected with miR‐1178‐siKD treated with actinomycin D (ActD) for the indicated periods of time. p53 mRNA levels were analysed by qRT‐PCR. (E–J) The mRNA and protein expression levels of p53 and CDK2 in both A549 cells and H1299 cells transfected with p53‐siKD were detected by qRT‐PCR and western blotting. (K–N) Transfection efficiency of CDK2‐oe was detected by qRT‐PCR and western blot in A549 and H1299 cells. Results are shown as mean ± SD of three biological replicates. **p < 0.01 and ***p < 0.001 versus indicated group.

Next, the downstream genes of miR‐1178 were explored; firstly, the DEGs in miR‐1178‐knockdown H1299 cells were analysed. As presented in Figure 5A, CDK2, PLIN5, TMEM174, and CAS expression were decreased after miR‐1178 knockdown, while p53, APOA4, DUSP8, and GSTT4 levels were upregulated. Further, the regulation of AMBRA1 expression on the level of expression of those 8 DEGs was verified by western blot assay. As presented in Figure 5B, overexpression of AMBRA1 significantly promoted p53 while inhibiting CDK2 expression. Based on the above results, p53 and CDK2 were selected as downstream genes of miR‐1178 for subsequent research. First, the effect of miR‐1178 on p53 and CDK2 protein expression was assessed by western blot assay. p53 expression was increased after miR‐1178 knockdown (*p* < 0.01), while its expression was decreased by miR‐1178 overexpression (*p* < 0.01). On the contrary, CDK2 level was downregulated by miR‐1178 silencing (*p* < 0.01) and was upregulated by miR‐1178 overexpression (*p* < 0.01, Figure 5C). Next, we performed a half‐time assay to detect the p53 mRNA remaining after the H1299 cells were transfected with miR‐1178‐siKD and treated with Act D for 1, 2, 3, 4, and 5 h. As presented in Figure 5D, the half‐time of the p53 transcript in H1299 cells was increased after miR‐1178 knockdown. Further, the regulation between p53 and CDK2 was explored; CDK2 expression was found to be increased after p53 silencing in A549 and H1299 cells (*p* < 0.001, Figure 5E–N). Therefore, a negative regulation between p53 and CDK2 was confirmed in NSCLC cells.

### 3.4 p53/CDK2 Mediated the Suppressive Effects of AMBRA1 Overexpression on NSCLC Cell Proliferation and Invasion

**FIGURE 6:**
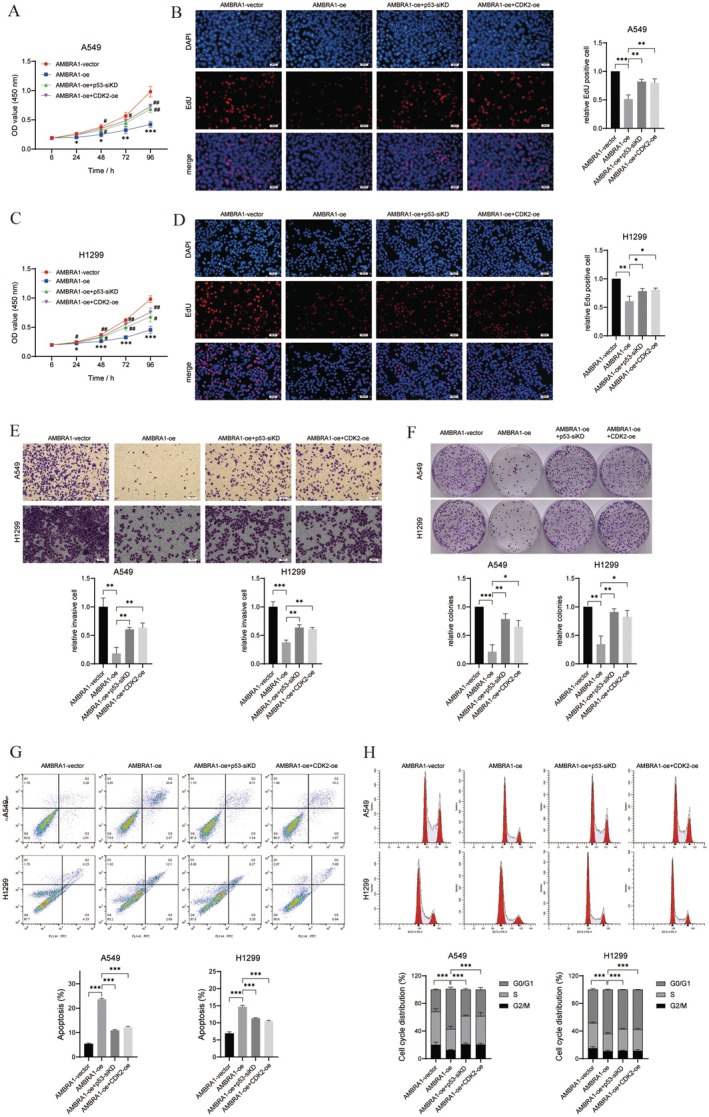
AMBRA1 decreased NSCLC cell proliferation and migration through promoting p53 expression and decreasing CDK2 expression. A549 and H1299 cells were co‐transfected with AMBRA1‐oe and p53‐siKD or CDK2‐oe, (A, C) Cell viability was detected by CCK‐8 assay, (B, D) EdU assay was conducted to observe cell proliferation, (E) cell invasiveness was determined by transwell assay, (F) cell colony formation ability was demonstrated with colony formation assays; for A549 cells, the number of colonies in the AMBRA1‐vector group was 469, in the AMBRA‐oe was 105, in the AMBRA1‐oe + P53‐siKD was 403, and in the AMBRA1‐oe + CDK2‐oe was 361. As for H1299 cells, the vector group formed 476 colonies, the AMBRA1‐oe formed 219 colonies, the AMBRA1‐oe + P53‐siKD formed 405 colonies, and the AMBRA1‐oe + CDK2‐oe formed 433 colonies. (G, H) flow cytometry was conducted to determine cell apoptotic rate and cell cycle distribution. Results are shown as mean ± SD of three biological replicates. *p < 0.05, **p < 0.01 and ***p < 0.001 versus indicated group or AMBRA1‐vector; #*p* < 0.05 and ##*p* < 0.01 versus AMBRA1‐oe group.

Further, the role of p53 and CDK2 expression in the inhibition of malignant progression of NSCLC cells by AMBRA1 overexpression was assessed. CCK‐8, EdU, transwell, and colony formation assays demonstrated that both p53 knockdown and CDK2 overexpression partially attenuated the inhibitory effect of AMBRA1 overexpression on cell viability, proliferation, and invasion (*p* < 0.05, *p* < 0.01 and *p* < 0.001, Figure 6A–F). As expected, the promoting role of AMBRA1‐oe on cell apoptosis was also partially reversed by p53 inhibition and CDK2 overexpression (*p* < 0.001, Figure 6G). Similarly, p53 knockdown and CDK2 overexpression partially suppressed AMBRA1 overexpression‐induced G0/G1 phase arrest (*p* < 0.001, Figure 6H). Therefore, AMBRA1 overexpression inhibited NSCLC cell proliferation, migration, and cycle progression through promoting p53 expression and decreasing CDK2 expression.

### 3.5 AMBRA1 Overexpression Suppressed the In Vivo Tumour Growth Through Downregulating miR‐1178

**FIGURE 7:**
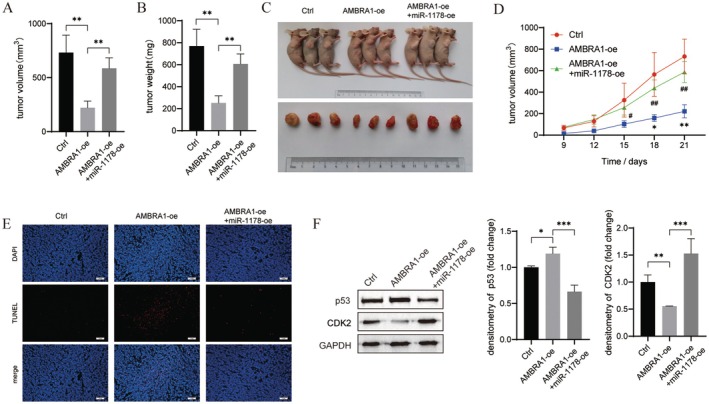
AMBRA1 inhibited in vivo tumour growth by decreasing miR‐1178 expression. Nude mice were given subcutaneous injections of H1299 cells containing a stable overexpression of AMBRA1 or both AMBRA1 and miR‐1178; tumour volume (A), tumour weight (B) and tumour growth curves (C, D) were measured. (E) Apoptotic cells in tumour tissues were analysed by TUNEL assay. (F) p53 and CycD expression in tumour tissues were confirmed by western blot. Results are shown as mean ± SD of three biological replicates. *p < 0.05, **p < 0.01 and ***p < 0.001 versus indicated group or AMBRA1‐vector; #*p* < 0.05 and ##*p* < 0.01 versus AMBRA1‐oe group.

To verify the effect of AMBRA1 and miR‐1178 expression on tumour growth in vivo, we performed xenograft experiments in nude mice. In comparison to control mice, the AMBRA1 overexpression group inoculated in nude mice had reduced tumour volume and weight (*p* < 0.05 and *p* < 0.01). While the inhibitory effect of AMBRA1‐oe on tumour growth was weakened by miR‐1178 overexpression (*p* < 0.05 and *p* < 0.01, Figure 7A–D). Also, the results from the TUNEL assay revealed that miR‐1178‐oe partially inhibited AMBRA1‐oe‐induced tumour cell apoptosis (Figure 7E). The western blot results in tumour tissues revealed that miR‐1178 overexpression suppressed AMBRA1‐oe‐induced p53 expression (*p* < 0.05 and *p* < 0.001) and promoted AMBRA1‐oe‐caused CycD inhibition (*p* < 0.01 and *p* < 0.001, Figure 7F).

## 4 Discussion

AMBRA1 is a disordered protein with strong plasticity and multiple biological functions [22]. AMBRA1 regulates multiple pathological processes in cancer cells, but the specific role it plays depends on the tumour microenvironment and genetic background, indicating that it can act as an oncogene or a tumour suppressor gene [23]. In lung cancer, loss of AMBRA1 promoted growth of mice lung adenocarcinoma (LUAD), and low levels of AMBRA1 are correlated with LUAD patients' poorer survival [11, 12]. In our study, AMBRA1 overexpression suppressed NSCLC cell proliferation and invasion (Figures 1 and 2), as well as in vivo NSCLC tumour growth (Figure 7), which was consistent with previous research. However, Liu et al. found that knockout of AMBRA1 in A549 cells attenuated TGF‐β‐induced EMT, suggesting AMBRA1 acts as a tumour suppressor gene. That is, its functionality is context‐dependent.

Recently, the role of miR‐1178 as a tumour modulator has been identified in multiple human cancers. In nasopharyngeal carcinoma [24], bladder cancer [25, 26], and pancreatic cancer [27, 28], miR‐1178 exerted oncogenic roles in increasing tumour cell growth and metastasis. While in hepatocellular carcinoma (HCC) [29], gastric cancer [30] and breast cancer [31, 32] miR‐1178‐3p was a tumour suppressor in HCC tumour growth and metastasis. As in NSCLC, Xu et al. demonstrated that miR‐1178 expression was upregulated in NSCLC tissues, and miR‐1178 mimics facilitated H1650 cell proliferative, migratory, and invasive capabilities [33]. Likewise, our sequencing data indicated that miR‐1178 was downregulated in AMBRA1‐overexpressed H1299 cells (Figure 3). Rescue experiment results confirmed that miR‐1178 overexpression partially weakened the suppressive effect of AMBRA1 overexpression on the cell proliferation, invasion, and cycle progression of A549 and H1299 cells (Figures 3 and 4), and xenograft tumour growth in vivo (Figure 7). Therefore, in the present study, miR‐1178 acted as an oncogene in NSCLC cells, which was consistent with the results of Xu et al. [33].

Further, the downstream genes of miR‐1178 were explored; sequencing data showed that CDK2 was downregulated while p53 was upregulated in miR‐1178‐knockdown H1299 cells (Figure 5). CDK2 is one of the core regulators of the cell cycle, which is active from the late G1 phase and throughout the S phase [34]. CDK2 can directly cause phosphorylation and inactivation of the retinoblastoma family proteins, leading to the release of the transcription factor E2F, which initiates the cell division cycle and prepares the cell for DNA replication [35]. CDKs are over‐activated in cancer cells, leading to uncontrolled cell proliferation [36]. AMBRA1 has been identified to play a vital part in the ubiquitination and proteasomal degradation of D‐type cyclins [11, 12, 13]. Simoneschi et al. [37] demonstrated that the deletion of AMBRA1 promoted the formation of D‐type cyclins in complex with CDK2, thereby reducing sensitivity to CDK4/6 inhibitors. Therefore, the AMBRA1‐CDK2 pathway is a key cell cycle regulatory mechanism and is closely related to genome stability in tumorigenesis. In the present study, CDK2 expression was downregulated by both AMBRA1 overexpression and miR‐1178 knockdown (Figure 5). CDK2 overexpression partially decreased the suppressive roles of AMBRA1 overexpression on NSCLC cell proliferation and invasion, and the facilitating role on cell apoptosis and cell cycle arrest at the G0/G1 phase (Figure 6). Therefore, AMBRA1 decreased CDK2 expression by inhibiting miR‐1178 expression, which ultimately suppressed tumour growth and metastasis of NSCLC.

p53 is an essential tumour suppressor and is the mutated gene most commonly found in human cancers. p53 mutations not only impair its anti‐tumour activity, but also confer oncogenic properties to mutant p53 proteins [38]. p53 remains an attractive target for cancer therapy. Over recent years, research has indicated that p53 is also implicated in the regulatory control of miRNA; that is, p53 modulates the transcription and processing of miRNA, and miRNA regulates the activity and expression of p53 [39]. Several core miRNA‐encoding genes that mediate the actions of p53 have been identified, such as the miR‐200 and miR‐34 families [39]. In this study, it was found that miR‐1178 could bind directly to the p53 3'UTR and regulate the expression of p53 negatively. Further rescue experiments demonstrated that p53 silencing partially decreased the suppressive function of AMBRA1 overexpression on NSCLC cell proliferation and invasion (Figure 6). Therefore, an AMBRA1‐miR‐1178‐p53 axis was implicated in the regulatory control of NSCLC cell proliferation and invasion. Besides, negative regulation existed between p53 and CDK2 (Figure 5). p53 directly affects cell cycle regulation; in detail, p53 promotes DNA repair by causing cell cycle arrest at the G1 checkpoint, allowing sufficient time for the repair of damaged DNA before it is passed on to daughter cells [40]. In addition to increasing p53 protein levels, DNA damage also upregulates p21 protein, which binds to and inhibits CDK2 kinase [41]. CDK2 kinase was found to phosphorylate p53 and activate downstream signal transduction pathways [42]. Hence, AMBRA1, through regulating cell cycle‐related proteins, CDK2 and p53, suppresses cell proliferation and invasion, meanwhile promoting cell cycle arrest and cell apoptosis.

## 5 Conclusion

In conclusion, AMBRA1 inhibited NSCLC cell proliferation and invasion, and increased cell apoptosis and G0/G1 phase‐cell cycle arrest through downregulating miR‐1178 expression. After knockdown of miR‐1178, p53 level was upregulated, while CDK2 was downregulated in NSCLC cells. More importantly, both p53 inhibition and CDK2 overexpression partially decreased the suppression effect of AMBRA1 on cell proliferation and invasion. Therefore, AMBRA1 suppresses the malignant phenotype of NSCLC cells by modulating the miR‐1178‐p53‐CDK2 signalling pathway.

## Author Contributions

**Jing Feng:** conceptualization (equal), methodology (equal), writing – original draft (equal), writing – review and editing (equal). **Shan Li:** data curation (equal), investigation (equal), writing – review and editing (equal). **Laihua Li:** formal analysis (equal), writing – original draft (equal), writing – review and editing (equal). **Zhiqiang Du:** conceptualization (equal), data curation (equal), writing – review and editing (equal). **Guangying Yang:** methodology (equal), writing – original draft (equal), writing – review and editing (equal). **Zhi Zhao:** methodology (equal), resources (equal), writing – review and editing (equal). **Xueke Fan:** data curation (equal), resources (equal), writing – review and editing (equal). **Na Wang:** data curation (equal), investigation (equal), writing – review and editing (equal). **Zhigang Zhao:** conceptualization (equal), data curation (equal), methodology (equal), writing – original draft (equal), writing – review and editing (equal).

## Ethics Statement

Experiments using mice were performed in accordance with the National Institute of health's Guide for the Care and Use of Laboratory Animals and were approved by Zhengzhou Yihe Hospital Affiliated to Henan University.

## Consent

The authors have nothing to report.

## Conflicts of Interest

The authors declare no conflicts of interest.

## Data Availability

The datasets used and/or analysed during the current study are available from the corresponding author on reasonable request.
